# Echocardiographic Demonstration  of the Effect of Varying Paced A-V Intervals on Ventricular Filling Pattern

**Published:** 2008-08-01

**Authors:** Damodar Kumbala, Sony Jacob, Masoor Kamalesh, Mithilesh Das

**Affiliations:** Krannert Institute of Cardiology, Indiana University, Indianapolis IN 46202

**Keywords:** A-V interval, ventricular filling, echocardiography

Impaired relaxation of the left ventricle (LV) (diastolic dysfunction)  invariably is associated with LV systolic dysfunction [1]. Atrial contribution to ventricular filling (second filling phase of diastole) is pivotal in the presence of impaired relaxation where early filling is reduced. Cardiac Resynchronization Therapy (CRT) has been shown to improve diastolic function in patients with heart failure [2,3]. Optimization of atrioventricular (AV) synchrony in sinus rhythm for patients with biventricular ICD (BiV ICD) for CRT is crucial in maximizing hemodynamic improvements [4,5].

In this brief report we demonstrate the use of Spectral Doppler to assess effect of varying paced atrioventricular (AV) intervals on the mitral filling pattern in a patient with  CRT for severe congestive heart failure with an  ejection fraction of 15% and a wide QRS interval.

## Case report

**Figure 1:**
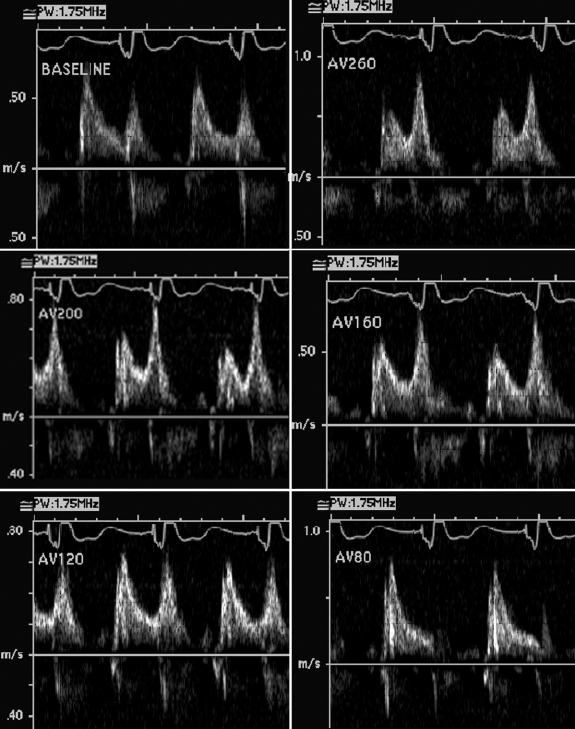
**a-f.** Mitral inflow pattern variation with changing AV intervals (in milliseconds). See text for details.

**Figure 2:**
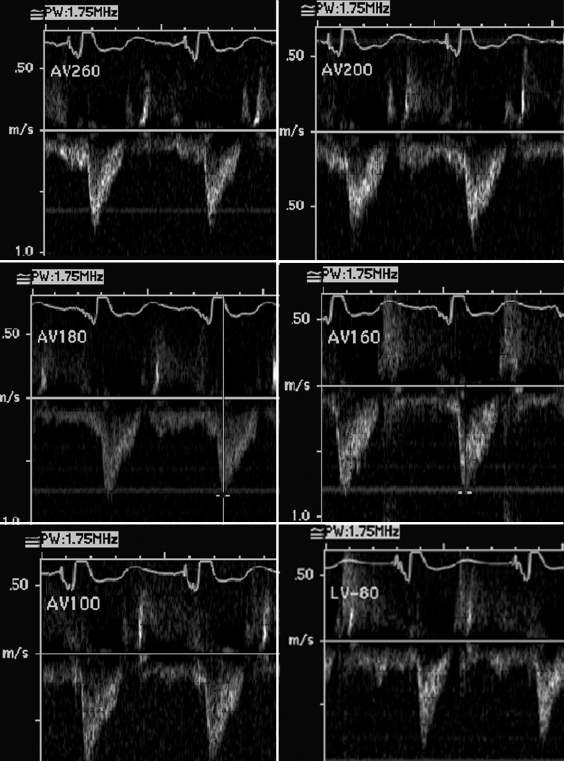
**a-f.** Left ventricular outflow Doppler profiles with changing A-V intervals (in milliseconds). See text for details.

A class 3 NYHA patient who underwent a BiV ICD implant was evaluated for optimization of CRT one month after implantation. This was performed by adjusting the AV interval and the LV to right ventricular (RV) pacing interval. The device interrogation revealed a complete AV block. The aortic valve velocity time integral (VTI) was recorded for cardiac output measurement while Doppler recording of A and E waves  demonstrated mitral valve inflow.   The above mentioned parameters were recorded by increasing the A sensed-V pace interval from 80 ms to 260 ms at increments of 20-40ms (Figure 1 and 2) 

After determining an optimal AV interval for the maximum aortic VTI and diastolic function (measured by finding the optimum E and A wave on Doppler recording), the paced LV to paced RV delay was tested. The optimum AV delay of 140 ms and an LV to RV paced delay was 30 ms to achieve the maximum cardiac output.

The effect of varying the AV interval on early and late diastolic filling of LV is shown in Figure 1.  At longer AV intervals (from 160-240 msec) there is relatively greater atrial contribution to ventricular filling  as shown by E/A ratio of <1. At AV interval of 140 the ratio begins to normalize; 120 msec interval being normalized. Reducing the interval further to 80 msec and 60 msec diminished atrial contribution significantly. The aortic velocity and VTI increased from 0.7 m/sec (VTI 18 cms) at longer AV intervals to 0.9 m/sec  (VTI 21.83) at optimal AV interval of 100-120 msec. (Figure 2)  The increase in aortic VTI is 17.5%. per cardiac cycle.

The patient's clinical symptoms improved during a two-week follow up. The NYHA class improved from III to II, with symptomatic improvement of shortness of breath as well as pedal edema.

## Discussion

The use of spectral Doppler echocardiography to optimize the effects of AV intervals on cardiac output following implantation CRT device is demonstrated here. Spectral Doppler interrogation of mitral inflow is easy to perform and is part of a routine echocardiographic study. The technique is widely available and easy to interpret. To maximize hemodynamic improvement, one should, not only maintain the AV synchrony but also optimize AV interval using echocardiography. This can ensure appropriate mitral filling.

Patients with diastolic dysfunction are particularly dependent on atrial contribution to ventricular filling. To  prevent any atrial input compromise after pacemaker implantation, mitral inflow can be interrogated with spectral Doppler to set the optimum AV interval.
